# Influences on indoor environmental trigger remediation uptake for children and young people with asthma: A scoping review

**DOI:** 10.1111/hex.13670

**Published:** 2022-12-07

**Authors:** Grace Lewis, Linda Milnes, Alexandra Adams, Jürgen Schwarze, Alistair Duff

**Affiliations:** ^1^ School of Healthcare, Faculty of Medicine and Health University of Leeds Leeds UK; ^2^ Asthma UK Centre for Applied Research, USHER Institute University of Edinburgh Edinburgh UK; ^3^ Paediatric Respiratory Unit Leeds Children's Hospital Leeds UK; ^4^ Child Life and Health, Centre for Inflammation Research The University of Edinburgh Edinburgh United Kingdom

**Keywords:** allergic sensitisation, asthma, asthma triggers, behavioural influences, children and young people, parent‐carer, scoping review

## Abstract

**Introduction:**

Children and young people (CYP) with asthma can benefit from reduced exposure to indoor environmental allergens and triggers but may not consistently have avoidance strategies implemented. To inform future interventions to increase trigger and allergen avoidance and enhance asthma control, a greater understanding of the influences on avoidance behaviours is necessary.

**Methods:**

A systematic scoping review was selected to summarize evidence on what influences family uptake of indoor environmental asthma trigger avoidance strategies for CYP with asthma and identify research gaps. Primary studies of any design, including CYP (≤18 years) with asthma, and/or parent‐carers, available in English and conducted since 1993, were eligible. Searches included nine databases, hand‐searching reference lists and citation searching.

**Findings:**

Thirty‐three articles were included and are summarized narratively due to heterogeneity. Influences appear complex and multifactorial and include barriers to strategy uptake, health beliefs and personal motivation. Research specifically related to family understanding of allergic sensitisation status and exposure risks, and how these may inform avoidance implementation is required. Patient and public involvement (PPI) was not reported in included articles, although two studies used participatory methods.

**Conclusion:**

There is limited research on family asthma trigger management, particularly what influences current management behaviours. Variation in families' ability to identify important triggers, understand exposure risk and consistently reduce exposures warrants further exploratory research to explain how families reach avoidance decisions, and what future interventions should aim to address. Further PPI‐informed research to address such gaps, could enable theory‐based, person‐centred interventions to improve the uptake of asthma trigger remediation.

**Patient or Public Contribution:**

An asthma‐specific PPI group contributed to the decision‐making for the funding for the wider project this review sits within. The findings of this scoping review have informed the subsequent phases of the project, and this was discussed with PPI groups (both adult and CYP groups) when proposing the next phases of the project.

## 1 INTRODUCTION

Asthma is a complex, heterogeneous, chronic airway condition, affecting more than one million children and young people (CYP) in the United Kingdom, and contributes to substantial economic and emotional burdens.^1^ Attempts to support CYP and families include self‐management programmes, which are multifaceted with medicating, monitoring and managing asthma triggers seen as core components.^2^ Physical asthma triggers can be broadly grouped as allergic, and irritant, and can be further subdivided into indoor and outdoor exposures. The focus of this review will be indoor environmental triggers including irritants and allergens.

There are potentially multiple indoor environmental triggers and exposure has been associated with increased asthma severity, exacerbations and reduced quality of life in CYP.^3^ Whilst intervention trials aim to reduce allergen presence in homes, including house‐dust mites (HDM), and pet allergens, many methods are not recommended for all by clinical guidance, in the United Kingdom. This is due to limited evidence for HDM exposure reduction methods,^2^ the complexity and heterogeneity of trials of reduction methods and subsequent challenges of aggregating data for systematic reviews or meta‐analyses for HDM and furry pet allergen reduction.^4^ Thus, trigger‐management advice is often to remove or avoid trigger sources, such as pets. However, longitudinal epidemiological evidence suggests that having a family member with asthma (without co‐existing rhino conjunctivitis) is not associated with pet withdrawal and does not deter pet acquisition.^5^ Moreover, a multicentre study conducted in 22 countries, demonstrated that adults with asthma and/or allergy, who owned pets and subsequently had children with an asthma diagnosis continued to keep pets, although with greater avoidance of cats than dogs or birds.^6^ Potential recall and selection bias were acknowledged in both studies.^5^, ^6^

Systematic reviews of asthma‐trigger education programmes have shown some promising outcomes. However, these are limited due to bias in included studies,^7^ a scarcity of eligible studies, and heterogeneous outcome measures further limited conclusions regarding intervention effectiveness.^8^ A more recent systematic review of educational interventions for CYP with asthma in underserved communities or minority groups, noted a lack of theory use and consideration of health literacy in intervention trials. Authors suggested greater attention be given to the beliefs and attitudes of those whose behaviour the interventions are designed to change.^9^

Despite healthcare providers giving avoidance advice, clinicians anecdotally note that families report continued exposures, particularly regarding pets they are emotionally attached to,^10^ and reluctance to rehome pets can lead to reluctance to suggest this.^11^ Multiple HDM reduction methods exist with varying levels of evidence to support their promotion for use in the homes of people with asthma.^4^ Given the aforementioned complexities surrounding HDM reduction method effectiveness measures, practical patient‐specific advice has been advocated, instead of relying on meta‐analyses of intervention trials.^4^, ^12^ However, little is known about whether families implement these measures, and how they choose between methods or barriers they may encounter, in real‐life settings. Avoidance of other indoor environmental exposures, such as environmental tobacco smoke (ETS) is advocated for general health,^13^ asthma control and primary prevention.^2^ How families actually manage indoor environmental asthma triggers outside of trial settings is not well described and long‐term intervention effectiveness also depends upon adherence to such health advice. Understanding adherence to supported self‐management plans by CYP and their families is complex since these include monitoring asthma, taking medications and managing asthma triggers with health‐provider support and to date, the literature focuses heavily on asthma medication adherence challenges. To enable the development of future interventions to address the apparent gap between clinical advice and environmental trigger avoidance uptake, a clearer understanding of the influences on avoidance and nonavoidance behaviours is needed. Furthermore, there is consensus that interventions should build from an evidence‐based understanding of the target problem or behaviours and context, in addition to careful selection and use of theory from early stages and iteratively throughout intervention development.^14^

### 1.1 Objectives and justification for selecting a scoping review

The objective of this scoping review is to describe what is known about CYP and/or parent‐carer beliefs, motivations and other influences involved in the uptake of avoidance of indoor environmental asthma triggers, in homes with a CYP with an asthma diagnosis. Additionally, the review aimed to discover evidence gaps. The overarching objective of the scoping review was to ascertain whether there is sufficient evidence to inform the development, or adaptation of a behavioural intervention to address continued exposures in CYP with moderate‐severe asthma and co‐existing allergic sensitisation* particularly to pets and/or HDM (*the presence of a positive reaction to allergens on testing, showing that there is an immune response mediated by exposure to the specific allergen. The immune response leads to airway inflammation and asthma symptoms and/or suboptimal control of asthma). Early literature searching to clarify the ideal type of literature review for these purposes suggested there is scant research into influences on asthma trigger avoidance behaviours. This led to the decision to select a scoping review to provide a high‐level overview of what is known and to identify research gaps.^15^

## 2 METHOD

This scoping review was guided by a seminal framework,^16^ alongside recent guidance.^15^, ^17^, ^18^, ^19^ These include review question development and study identification through database searching, study selection, charting or synthesizing and disseminating findings.^16^ A priori protocol was written as recommended.^15^, ^20^

### 2.1 Scoping review questions

(1) What is known about CYP and parent/carer beliefs regarding indoor environmental asthma triggers in homes?
(2) Do their beliefs inform exposure reduction strategy uptake?
(3) What factors influence avoidance/nonavoidance behaviours or adherence to avoidance advice?
(4) Are CYP/parent‐carers motivated to reduce environmental trigger exposures at home, and what may further motivate avoidance?
(5) Are there any relevant research gaps which may require attention before further behavioural intervention development or adaptation?

### 2.2 Search strategy

Search terms were developed according to participants, concepts and contexts of interest^19^: asthma AND/OR allergic sensitisation AND triggers AND children AND/OR parent/carers AND beliefs AND/OR behaviours AND qualitative OR quantitative OR mixed methods. A search string is available in Supporting Information: 1. Table 1 details the inclusion/exclusion criteria, including reasons for the selection of participants, concepts and contexts of interest for the scoping review.

Terms were refined following an initial search using Ovid Medline, and relevant synonyms, mesh terms and headings were used. Final terms were extended to Embase, CINAHL, PsychINFO, Google scholar and Cochrane Database. Grey literature databases searched included Zetoc, OpenGrey and Ethos. Systematic reviews were not included in the scoping review but were read and reference lists were reviewed for relevant primary studies. Citation searching was conducted using Scopus, Google Scholar and Web of Science, and reference lists of key and included articles were searched. This strategy was developed to capture broader studies of self‐management that included data related to influences on asthma trigger avoidance strategy uptake.

Searches were limited to articles available in English and those with primary, empirical data collected and published since 1993. This reflected that British Thoracic Society guidance changed in 1993 to include asthma trigger avoidance advice.^21^ Further inclusion/exclusion criteria are detailed in Table 1. These restrictions allowed a balance between relevance and search breadth.^18^, ^22^ Initial searches were run from January to March 2020 and updated in August 2021. Database alerts were used throughout to track additions to the literature with matching search terms. Deduplication within databases was conducted where available, and further deduplication was recorded after importations to Mendeley.

**Table 1:** Inclusion/exclusion criteria

Restriction area	Inclusion	Exclusion	Explanation
Study design	Any primary study design highlighting beliefs and opinions about asthma triggers (concept of interest) in CYP and/or parents/carers of children with asthma and trigger avoidance strategies	Studies designed to evaluate effectiveness of an intervention. However, if baseline measures were taken to establish beliefs before an intervention, these could be included if they could be extracted in isolation	The aim of the scoping review is to understand whether triggers are noted and/or avoided by CYP/parents under usual care, rather than those who have undergone an intervention trial. Incorporating all designs allowed for broad evidence scoping
Studies exploring other triggers	Those including indoor triggers, in any country (context) where findings relating to these can be extracted separately	Studies exploring only beliefs around psychological triggers or outdoor environmental triggers	Numerous studies were noted exploring only psychological or outdoor triggers on developing and piloting search strategies
Participants	CYP (under 18 years) or parents/caregivers of CYP with asthma or asthma and co‐existing allergic sensitisation—if reported	Adult only participants with asthma or unclear descriptions of diagnoses (e.g., wheeze rather than asthma). Studies including only those under the age of 5 years/parents of under 5s with asthma, were ineligible	Due to differences in asthma and asthma management between adults and those under 18 years.^23^, ^24^ Asthma is difficult to diagnose in under 5s.^2^, ^25^

Abbreviation: CYP, children and young people.

### 2.3 Data extraction

Two reviewers (G. L. and L. M.) conducted article selection and data extraction. Data extraction followed scoping review guidance,^16^ and further details included study aims, dates of data collection (where available) and confirmation of ethical approval. A copy of the data extraction table is available via the protocol.^20^

## 3 RESULTS

### 3.1 Article retrievals

**Figure 1:**
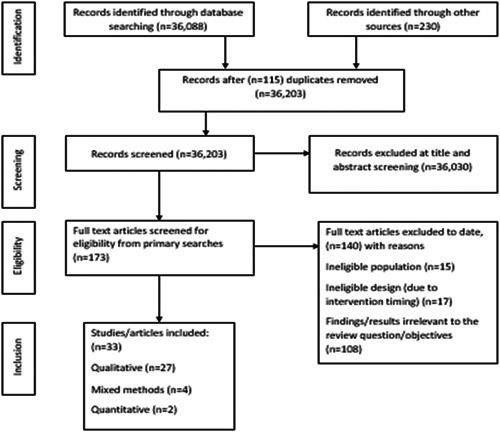
PRISMA flow diagram

Electronic database searching retrieved 36,088 articles and a further 230 were retrieved through hand‐searching reference lists and database citation searching. After deduplication, 36,203 were screened and 36,030 were excluded based on title or abstract. Full‐text screening was conducted for 173 articles and 33 were included. Reasons for exclusion include ineligible populations/participants (*n* = 15), ineligible study design, due to intervention timing (*n* = 17), or results that were not suited to answer the scoping review questions (*n* = 108). Retrieval results are displayed in Figure 1: PRISMA flow diagram and a PRISMA‐ScR checklist^26^ is available in Supporting Information: 2.

### 3.2 Study designs

Of the 33 studies, 27 were qualitative, two quantitative and four mixed methods. Methodologies and methods employed are detailed in Supporting Information: Table 2 (Supporting Information 3) alongside study aims, context/setting, participants, and study designs. Due to the heterogeneity of included studies, narrative findings are presented. Due to the broad scoping nature of this review, included studies also reported findings that are outside the scope of this review. For clarity, only the findings or results that are pertinent to this review are reported.

### 3.3 Narrative summary of findings

#### 3.3.1 Ability to identify triggers

A family's ability to identify triggers is an important step preceding decisions about avoidance strategy implementation. Participants were able to identify some potential indoor environmental triggers across the majority of included studies.^27^, ^28^, ^29^, ^30^, ^31^, ^32^, ^33^, ^34^, ^35^, ^36^, ^37^, ^38^, ^39^, ^40^, ^41^, ^42^, ^43^, ^44^, ^45^, ^46^, ^47^, ^48^, ^49^, ^50^, ^51^ A mixed methods study reported that 77% of 200 parents of 5–12‐year‐olds with asthma, avoided some indoor environmental triggers suggesting recognition, although most identified ETS and dust.^52^ Parents and CYP with asthma were asked to rank triggers by impact in an American study with socioeconomically disadvantaged families; ETS, dust and cockroaches were believed to have the highest impact, followed by pets, mould and dry heat.^49^

Trigger identification was not universal across studies. An English qualitative study of 11–18‐year‐olds with severe, uncontrolled asthma, reported that participants had very limited trigger knowledge; whilst most were aware of ETS as a trigger, many lived with a pet but were unaware pet allergens could trigger asthma or denied that pet exposure led to symptoms, even where participant descriptions suggested otherwise.^37^ Another qualitative study from England described parents' belief that some CYP with asthma were unable to identify triggers themselves.^50^ Authors of a grounded theory study conducted in America noted some families were not able to recognise which triggers led to exacerbations and such uncertainty resulted in anxiety.^53^

#### 3.3.2 Trigger information provision and experiences with triggers

Receipt of trigger information was identified as a potential factor influencing avoidance strategy implementation in a mixed methods pilot study that investigated parents' trigger knowledge and strategy uptake^39^: African American parents (*n* = 4, of *n* = 12 participants) reported having not received trigger information from healthcare providers, in contrast to the eight White participants. This was reflected in greater awareness of triggers (*t* = 2.43 *p* = .017) and higher uptake of avoidance strategies (*t* = 1.98; *p* = .04; particularly for HDM reduction *t* = 3.23; *p* = .009) in White families with a child with asthma. Although, all were aware of ETS and pets as triggers. Those who had not received trigger avoidance advice were less trusting of healthcare providers and were less likely to report having discussed asthma with others or sought information themselves. However, due to this being a pilot study, the results were deemed provisional.^39^

A qualitative study with White British and British South Asian families with a child with asthma, also highlighted that across ethnicities families reported they had not received an asthma action plan, in which families are invited to note triggers. Furthermore, those who did not speak English as a first language experienced additional barriers where information was not provided in their first language. Although, all families experienced problems with accessing or understanding information, including trigger information.^46^

For fathers in a Canadian qualitative study, trigger advice from other parents of children with asthma was valued and observing an exacerbation following trigger exposure led to trigger recognition.^54^ This was echoed in a Norwegian study of 15 children (7–10 years) who learned to recognise triggers through previous exacerbations or allergic reactions and at times endured continued exposures or continued activities that left them feeling exhausted, to maintain social normality.^55^

Findings from two North American qualitative studies reported that participants who were unable to identify triggers described that they did not know the information they needed to enable identification.^44^, ^53^ A qualitative study in the United States described parents being overwhelmed when multiple trigger exposures were possible, and there was uncertainty in 9 of their 10 participants about risks attributable to triggers.^56^ Younger children (7–12‐year‐olds with moderate‐severe asthma), in the United States, were able to identify triggers such as a family pet and attribute coughing to exposure, but rarely knew how to avoid triggers.^29^

Two articles mentioned that where trigger exposure also led to noticeable allergic symptoms, such as facial oedema, triggers were more easily recalled,^51^ by children as young as 7 years old.^55^ In contrast, some parents did not consistently notice signs of allergy or deteriorating asthma control.^44^ Parent participants in a study in Taiwan described that they did not know their children's triggering allergens and that their 8–12‐year‐olds should be aware themselves. This contrasted parental beliefs that they should help CYP with asthma control, in the same study. Parents also experienced difficulty differentiating colds, asthma and allergic rhinitis symptoms.^42^

HDMs were mentioned far less than other triggers. One article reporting a qualitative study with mothers of children with asthma in Australia noted that HDM had to be identified as a trigger and explained by health professionals and that not knowing this sooner led parents to reassess their competence after an exacerbation and led to feelings of guilt.^31^

#### 3.3.3 Myths and misconceptions

Misconceptions and myths recurred in accounts of participants' beliefs. Asthma was believed to be episodic rather than chronic with symptomatic episodes by some participants.^44^ There was also confusion between perceived asthma aetiology and asthma symptom triggers. For instance, parents believed asthma only occurred when CYP were exposed to triggers,^46^ such as dusty schools.^33^ Such misconceptions led parents to believe that asthma could be cured by trigger eradication.^44^ Parents also believed that whilst dust should be minimized, children were likely to outgrow asthma and ‘willpower’ could limit the likelihood of chronicity.^43^ ^,p.134^

#### 3.3.4 ETS beliefs and experiences

Despite broad recognition of ETS as an asthma trigger, some studies highlighted misconceptions and risk‐taking. Some parent‐caregivers in an American qualitative study believed ETS exposure could enable tolerance.^41^ Some CYP reported experimenting with cigarettes, despite knowing the risks.^37^, ^47^ Conversely, in some studies, CYP noted that parent‐carers continued to expose them to tobacco smoke.^32^, ^33^, ^40^ CYP explained this by noting cultural norms and the unacceptability of requesting guests to smoke outdoors in one study.^33^ Parents also reported feeling they lacked control over the presence of pets and smokers,^28^ but the underlying reasons for limited control were not clear. Barriers to tackling this appeared related to parental health beliefs, personal and environmental circumstances rather than socioeconomic limitations, such as healthcare access and medical insurance coverage, as often presumed in low‐income groups, as sampled in this study.^28^ Some teenagers became able to self‐advocate ETS avoidance at home either by removing themselves from the area or requesting parents smoke outside.^45^ In contrast, CYP sometimes avoided confrontation with others over ETS exposure by moving away or using reliever inhalers.^32^

#### 3.3.5 CYP age

Age has also been identified as a factor in CYP taking responsibility for asthma self‐management.^41^, ^42^ A qualitative study with parent‐carers of teens (14–18 years), with asthma, reported parent‐carers believed that age was a suitable measure of when CYP could take responsibility, and 14–18 years was an appropriate age; one exception was a parent of a teenager with learning difficulties.^41^ However, whilst teenagers were keen to mitigate their asthma diagnosis as they moved into adulthood, few noted trigger avoidance in their mitigation strategy in an American study.^45^

#### 3.3.6 Avoidance strategies noted by participants and influences on strategy uptake

Whilst beliefs and perceptions likely influence strategy uptake, other issues were apparent that suggested simple information provision may not lead to uptake. For example, a cross‐sectional survey of American parents (*n* = 638) of CYP (aged 3–16‐year‐olds with asthma), showed there was no association between previous trigger education (written or discussions in clinic) provision and exposure to triggers in the home. However, dog ownership was associated with lower parental education levels (odds ratio [OR]: 2.3; 95% confidence interval [CI]: 1.2–4.3). Similarly, household smoking was associated with low income (OR: 1.9; 95% CI: 1.0–3.7) and low parental education levels (OR: 4.5; 95% CI: 2.4–8.2). Also, there was no association between exposures and asthma symptoms but the authors did not control for medication use or inhaler technique.^57^

In a mixed methods study of 200 parent‐caregivers of 9–12‐year‐olds with asthma, 77% described avoiding some triggers. However, when questioned about specific triggers, avoidance reports were low for pets (35%), tobacco smoke (29%), HDM (10%) and soft toy removal (14%; undertaken to reduce HDM exposures), and in qualitative interviews, increased cleaning, cleaning when children were not present and smoke‐free rules were most frequently reported.^52^

Few articles mentioned parents' use of air purifiers and dehumidifiers,^27^ with use, particularly on rainy days.^42^ Few articles mentioned HDM‐proof bedding,^31^, ^42^, ^51^ with one citing parents' uncertainty regarding effectiveness.^42^ Although some families reported the use of HDM‐proof bedding, they also suggested other strategies that may help (such as carpet removal) but had not yet implemented this,^51^ suggesting partial strategy implementation despite knowledge.

Two articles noted CYP knew that pets may trigger asthma and that this led to avoidance but that this was usually where CYP had other (non‐asthma‐related), allergic symptoms.^38^, ^50^ Partial avoidance strategies were also described, with participants disallowing pets into CYP's bedrooms.^44^, ^50^ However, how these strategic decisions were reached and their perceived effects on asthma control were not discussed.

An English sociological study of nine families suggested whilst most could identify some triggers, families did not always believe these were applicable. Families with pets either asked children to stay away from pets or made decisions to keep pets (e.g., rabbits) due to their child's emotional attachment.^58^ However, it was unclear whether rabbits were kept outdoors. Another family kept cats and dogs despite believing their child may be allergic and felt they mitigated risks by hand washing. A further family timed removal of soft toys for a ‘deep freeze’ (to mitigate HDM exposure) to avoid upsetting their child. Some families noted triggers but did not enforce avoidance as this was considered more unsettling to family life than asthma.^58^

#### 3.3.7 Inhaler use and trigger exposures

The use of reliever inhalers was mentioned in reaction to trigger exposures and for preparedness for potential exposures outside of the home.^45^ However, others described that despite knowledge about asthma triggers, CYP did not always carry reliever inhalers,^32^ and sometimes took risks related to triggers.^38^

#### 3.3.8 Motivation and trigger avoidance

One study described using ‘structured interviews’ based upon attribution theory, to investigate causal attributions participants applied to explain self‐management successes and failures. Both CYP (9–13 years) and parents‐carers attributed trigger avoidance success and failure to predominantly internal, and personally controllable reasons. Whilst triggers were referred to broadly, rather than by individual types of trigger or allergen in the article, motivation was discussed. Both intrinsic motivation (including being observant of triggers) and effort for self‐management were seen as causally related to self‐management successes and failures by participants. Children attributed their trigger avoidance success and failure to mostly internal (85.9%–96.9%) and controllable (73%–93.2%) but unstable (69.2%–79.4%) causes. Parents also believed causes of successful or failed trigger avoidance were internal (79%–68.3%), mostly controllable (85.5%–54%) but unstable (59.7%–73%). However, external issues also impacted participants, for example, some exposures appeared especially challenging to avoid due to their abundance (e.g., pollen).^59^ ^,p.276^

Whilst no included studies aimed to explore what might motivate increased trigger avoidance, some studies briefly discussed motivation as a barrier to improving asthma self‐management in their findings: Teenagers in a Swedish qualitative study were ambivalent about asthma self‐care, as they attempted to balance managing asthma with maintaining social norms.^48^ In an English qualitative study, some older teenagers described their indifference towards self‐care and parents reported teenagers' low motivation and risk‐taking behaviours as barriers to successful self‐management.^50^ However, one Canadian mixed methods study highlighted CYP's wish to learn from other slightly older adolescents with experience in managing allergies and asthma suggesting interaction may enhance self‐management.^38^

Other studies of asthma self‐management experiences suggested parents and/or CYP are often motivated to improve family management of asthma (including trigger management), but that other social, and familial challenges constrain the implementation of improvement strategies. A grounded theory approach describing the main concerns of 11–16‐year‐olds with asthma in Ireland, suggested CYP tested boundaries with trigger exposures and attempted to balance trigger management with engagement in activities with peers. However, CYP remained motivated to manage asthma.^47^ A further grounded theory study identified that self‐management involved families learning about symptoms and associated triggers and that they attempted to ‘catch the asthma before it got out of hand’.^27^ ^,p.359^ In contrast, older teenagers have acknowledged taking risks with known triggers and needing support to assess risks safely.^38^ Younger children (7–10 years) described known triggers but sometimes pushed themselves and ignored triggers to avoid appearing different or being harassed by peers.^55^

Following a qualitative study in England, a parental typology to describe asthma trigger management responses was developed. Parents were grouped as ‘preventors, reactors or compensators’: whilst all were motivated to preserve normality, the strategies and timing of implementation differed depending on whether parents attempted proactive, preventative trigger avoidance or compensated for exposures by implementing some exposure reduction strategies reactively or reacted to triggers only after an asthma exacerbation.^34^ ^,p.109^

#### 3.3.9 Other barriers to avoidance

Costs of HDM‐proof bedding were noted as a barrier to purchase by parents in one study.^33^ Studies reporting recruitment from low‐income groups or communities, identified other barriers to trigger avoidance strategy implementation. These included lack of control of overcrowding, financial constraints for pest control,^35^ challenges with controlling shared environments and landlord refusal to support tenants with resolving these issues.^36^ Parents in disadvantaged settings made as many environmental adaptions as possible (e.g., changing air‐conditioning filters). However, CYP identified and prioritised emotional triggers, including the threat of neighbourhood violence, where parent‐carers noted physical triggers.^49^ Similarly, fear of neighbourhood violence and poor outdoor air quality limited CYP's time spent outdoors and deterred increased ventilation by opening windows.^36^

## 4 DISCUSSION

This scoping review was undertaken to outline the extent of current evidence on the influences on indoor environmental trigger avoidance at home, for CYP with asthma. Most of the included articles took a broad view of asthma self‐management and explored many aspects beyond trigger management. This limits the extent to which the review questions could be answered in terms of detailed explanations of behaviours, yet this highlights research gaps. Three articles had aims focussed solely upon asthma triggers.^34^, ^39^, ^57^ These studies provided insight into parent typological responses to CYP's asthma triggers,^34^ the lack of association between advice to avoid triggers and parental uptake of avoidance^57^ and reported racial inequity of receipt of avoidance information in an American pilot study of 12 parents.^39^ However, all focussed on parent‐carer perspectives and did not include CYP as participants. Inclusion of CYP's perspectives could further understanding of the processes involved in strategy uptake decisions. Moreover, the processes involved in family decision‐making regarding trigger avoidance were touched upon in included articles, but detailed explanations of behavioural influences remain unclear. This scarcity of in‐depth, explanatory research on the topic is echoed in evidence syntheses of self‐management practices and experiences of parent‐carers of CYP with asthma,^60^ and barriers and facilitators for successful self‐management,^61^, ^62^, ^63^ which had a greater focus on medication adherence than trigger avoidance adherence. Whilst this is unsurprising, given the importance of medication in asthma management, it remains challenging to develop evidence‐based trigger exposure reduction interventions where current behaviours and behavioural influences remain unclear.

None of the included articles referred to the allergic sensitisation status of included participants (with exception of those with visible signs and symptoms of allergic reaction) or established whether sensitisation was understood and whether this may be related to avoidance strategy uptake. Although there is evidence of good parental recall of positive skin prick test results for allergen sensitivity, parents may not link exposures to aeroallergens (to which their child is sensitised) to acute asthma exacerbations.^64^ Some included studies noted that according to parents, children did not recognise symptoms of deteriorating asthma control,^59^ which for parents in one study, led to delayed asthma treatment.^42^ Suboptimal adherence to asthma monitoring has also been reported.^63^ Whilst an intervention to improve symptom and trigger recognition using home monitoring resulted in increased symptom recognition and trigger recognition, these increases were accompanied by a postintervention decrease in quality of life.^65^ ‘Being on alert’ to asthma triggers was noted in a study included in the scoping review,^27^ ^,p.361^ and others suggested this may increase the emotional burden of asthma management, through increased anxiety.^53^ These complexly linked issues warrant further consideration in future intervention development.

Only one study noted the emotional value of pet keeping despite being a suspected trigger. However, the children's sensitisation status had not been confirmed.^58^ Whilst evidence suggests few families (4.7%^6^; 8%^5^) rehome pets after advice to do so, greater clarity is needed to explain whether families understand the role of allergic sensitisation and related exposures in asthma control, as this may be a potential factor in pet‐keeping decisions and may be considered alongside emotional gains of pet‐keeping.

The included articles reporting ETS exposure at home as a trigger,^45^, ^50^ and first‐hand CYP smoking,^47^, ^50^ included some of the most recently published studies. Smoking and ETS are well‐established asthma triggers and have well‐known causative detrimental effects for CYP.^13^ Recent evidence showed an association between reduced asthma‐related hospital admissions and Scotland's Take it Right Outside smoke‐free home campaign,^66^ suggesting a plausible correlation between reduced exposure and reduced exacerbations. However, smoking prevalence remains disproportionately higher in disadvantaged UK homes,^67^ potentially placing CYP at risk of exposure. Thus, contemporary data for ETS exposure in homes of CYP with asthma remain important for the development of targeted interventions to reduce exposures. Environmental vapour from electronic cigarettes or similar devices has also emerged in surveys with adults and adolescents with asthma in the United Kingdom as a potential trigger,^68^ and maybe an area for further exploration amongst CYP with asthma who may be exposed.

### 4.1 Strengths and limitations

This review sought to provide a high‐level overview of what was known about beliefs, and other factors influencing avoidance of indoor environmental asthma triggers. However, there are many asthma triggers and some act in synergy, for example co‐existing viral infection and allergen exposure reduce asthma control and increase the risk of hospital admission.^69^ Greater understanding of family experiences and perceptions about such synergistic effects may be beneficial for the promotion of trigger avoidance interventions.

The main strengths of this review are the broad search strategy employed to minimise risks of missing relevant articles and the subsequent identification of research gaps. Although the review focus is indoor environmental triggers, CYP prioritised emotional triggers, where parent‐carers appeared to prioritise environmental triggers in one study^49^; whether this relates to strategy prioritisation or uptake is also of interest, particularly if families may not discuss triggers amongst family members, a factor which the included articles did not determine.

The search limitations applied were specific to the broader aims of the project this review sits within and sought to balance breadth with practicality, as guidance recommends.^18^ Limiting searches to English language full texts may have introduced language bias.^70^ However, where abstracts were available in English, there were none that would have necessitated full‐text translation, when assessed against eligibility criteria. Included studies were from high‐income countries, which may relate to exclusion of articles in other languages or may reflect other publication biases. Moreover, it may reflect the scarcity of evidence on the topic.

Most scoping reviews do not encompass quality appraisal,^71^ despite methodological debates over this and continued critique of appraisal absence.^17^, ^72^ The overarching aim was to establish whether there was sufficient evidence to describe factors influencing low uptake of avoidance strategies and had there been sufficient evidence, quality appraisal could have informed whether the evidence was robust enough to begin intervention development. However, due to the scarcity of explanatory evidence for current behaviours, it was concluded more research is needed. Consequently, this review has not included quality appraisal. Yet it is notable that no included studies reported patient and public involvement (PPI) although two used participatory methods.^40^, ^49^ Stakeholder consultation^16^ for this review was considered but not undertaken due to the project timelines, author expertise and use of a search strategy that sought only published empirical evidence.

## 5 CONCLUSIONS

Myths, misconceptions and challenges associated with trigger identification or risk attribution remain for some families and could inform avoidance strategy uptake. Families living with socioeconomic disadvantages often face additional barriers. For those able to identify triggers, and with access to medical advice, strategy uptake appears variable and sometimes partial, which appears to reflect the complexities of balancing other family demands by parent‐carers,^27^, ^34^, ^53^ and the CYP's desire to live lives close to those of their peers without asthma.^30^, ^47^, ^48^, ^55^ Failure to either implement or report the use of behavioural change theory for asthma trigger reduction intervention planning, development and implementation have been acknowledged.^7^, ^9^ Future research should aim to elucidate the influences on behaviours to inform the appropriate choice of behavioural theory for interventions. As intervention acceptability and effectiveness are maximised when they are ‘person‐based’,^73^ such research ahead of intervention development would benefit from PPI and in‐depth qualitative study. Further exploratory research focussed on family understanding of allergic sensitisation, indoor environmental asthma triggers related perceived asthma control, and what may motivate increased avoidance, are necessary to inform targeted family‐centred interventions applicable to home settings.

## AUTHOR CONTRIBUTIONS

Grace Lewis had significant involvement in the review design, extraction, and interpretation of data and the first and subsequent drafts of the review manuscript. Linda Milnes, Alistair Duff, Jürgen Schwarze and Alexandra Adams provided guidance for the design and delivery of the review. Linda Milnes provided guidance for Grace Lewis to conduct searches. Grace Lewis and Linda Milnes conducted article selection and data extraction according to a protocol agreed upon by all authors. Alistair Duff, Jürgen Schwarze and Alexandra Adams were consulted for any disagreements in the article selection or data extraction. All authors provided final approval for publication of the manuscript.

## CONFLICT OF INTEREST

The author declares no conflict of interest.

## Supporting information

Supporting information.

Supporting information.

Supporting information.

## Data Availability

No primary data are available to share.
