# Supplementary material for: Influences on indoor environmental trigger remediation uptake for children and young people with asthma: A scoping review
Source: Health Expect. 2022 Dec 7;26(1):87–97. doi: 10.1111/hex.13670 (PMC9854302; doi:10.1111/hex.13670)
Supplement: Supplementary file 1 — Supporting information. [file HEX-26--s002.docx]

1 (child or children or childhood or adolescen* or teen or teens or teenager* or youth or youths or girl or girls or boy or boys or p?ediatric* or juvenil* or "young people" or "young person" or "young adult*" or "schoolchild*").ti.

2 (child or children or childhood or adolescen* or teen or teens or teenager* or youth or youths or girl or girls or boy or boys or p?ediatric* or juvenil* or "young people" or "young person" or "young adult*" or "schoolchild*").ab. /freq=2

3 Pediatrics/

4 young adult/

5 child

6 adolescent/

7 child/

8 or/1-7 [young people]

9 exp asthma/

10 asthma*.ti.

11 asthma.ab. /freq=2

12 or/9-11 [asthma]

13 (dust or mite*).ti.

14 (dust or mite*).ab. /freq=2

15 (animal dander or pet* or dog* or cat*).ti.

16 (animal dander or pet* or dog* or cat*).ab. /freq=2

17 Allergens/

18 (aller* or hypersensitiv* or sensiti?ation).ti.

19 (aller* or hypersensitiv* or sensiti?ation).ab. /freq=2

20 (trigger* or exacerbat*).ab. /freq=2

21 (trigger* or exacerbat*).ti.

22 or/13-21 [triggers]

23 ((barrier* or facilitator* or behavio?r*) adj5 asthma*).tw.

24 ((challenge* or constrain* or experience* or motiv* or influenc* or chang* or enab* or attitude* or perception* or perceive* or belief* or believe* or opinion* or view* or standpoint*) adj5 asthma*).tw.

25 ((incent* or factor* or limit* or demand* or driver* or driving* or facilitat* or threat*) adj5 asthma*).tw.

26 (interview* or qualitative or theme* or survey* or questionnaire* or focus group*).tw.

27 health knowledge, attitudes, practice/

28 "Attitude of Health Personnel"/

29 exp *Qualitative Research/

30 focus groups/

31 interviews as topic/

32 exp *Questionnaires/

33 family/px

34 parent/px

35 *Caregivers/px [Psychology]

36 ((attitude* or perception* or belief* or experience* or perspectiv* or knowledge or understanding) adj3 (carer* or caregiv* or care giv* or parent* or mother* or father*)).tw.

37 Attitude to Health/

38 or/23-37

39 8 and 12 and 22 and 38
