# Supplementary material for: Influences on indoor environmental trigger remediation uptake for children and young people with asthma: A scoping review
Source: Health Expect. 2022 Dec 7;26(1):87–97. doi: 10.1111/hex.13670 (PMC9854302; doi:10.1111/hex.13670)
Supplement: Supplementary file 3 — Supporting information. [file HEX-26--s003.docx]

| **Reference** | **Context/country** | **Aim/purpose** | **Population** | **Study design** |
| --- | --- | --- | --- | --- |
| Crosland *et al,* (2009) [34] | Northeast England, UK | Provide an exploration of parental perceptions of indoor environmental asthma risks and outline strategies taken in relation to those risks | (n=22) parents of (n=32) CYP (aged 4-16 years) with asthma (n=22 mothers and n=2 fathers) | In-depth qualitative interviews analysed using constant comparative analysis. |
| Holley *et al,* (2018) [50] | Southampton & Isle of White, UK | To gain insight into barriers and facilitators for self-management, to inform an intervention | Adolescents (n=28), 16-18 years with asthma, and (n=12) parents | Semi structured qualitative interviews and focus groups were conducted and analysed using inductive thematic analysis. |
| Laster *et al*, (2009) [35] | Metropolitan Atlanta, USA | Inform intervention development | (Total participants n=28) CYP aged 8-17 years, with moderate-severe, asthma and their parents/caregivers. Most (22/28: 78.6 %) were described as urban, low income, Medicaid/SCHIP- State Children’s Health Insurance Program receivers. 7.1% were uninsured | Focus groups and thematically grouped description was used. |
| Finkelstein *et al,* (2002) [57] | USA | Assess prevalence of environmental trigger presence. Identify risk factors for trigger exposure. Determine whether parental trigger education is associated with fewer trigger exposures. | Parents of children (n=638 children) with asthma, aged 3-15 years | Cross-sectional study. Face-to-face interviews using ‘close ended questions’ [57, pp.259] were used to collect data. |
| Archibald *et al,* (2015) [44] | Urban Canada | Gain insight into parents’ asthma information needs | 21 Parents of (n=23) CYP with asthma | Qualitative semi-structured interviews. Interpretive description guided design and analyses. |
| Shaw & Oneal (2014) [53] | USA | To develop a grounded theory to guide interventions to reduce emergency visits and hospitalisations due to asthma | 10 families, made up of 20 participants (n=13 parents, n=7 CYP, aged 0-18 years) | Semi-structured interviews. Grounded theory design and analyses. |
| Jonsson *et al,* (2017) [48] | Sweden | Describe daily life experiences of adolescents with asthma, particularly relating to self-management. | 16-18-year-olds (n=10) | Semi structured qualitative interviews analysed with systematic text condensation. |
| Velsor-Friedrich, *et al,* (2004) [32] | USA (including four areas: inner-city, suburban, and rural) | Explore teens experience and behaviour related to self-management. | 14-18-year-olds (n=24) | An ethnographic approach to exploratory focus groups. |
| Cashin *et al,* (2008) [54] | Canada | Understand lived experience of being a father to a child with asthma | N=8 Fathers (of 7-11-year-olds with asthma) | Phenomenological, inductive design, using open interviews. |
| Gabe *et al,* (2002) [30] | West London, UK | Aimed to study the meaning of asthma amongst CYP and strategies used to ameliorate asthma impact. | N=55 (11-16 years old) CYPs’ data was analysed (14 parents were present in interviews, but not interviewed directly) | Semi-structured interviews were conducted. Ethnograph (software) was used for analysis. |
| Maltby *et al,* (2003) [31] | Perth, Australia | To outline the meanings mothers ascribe to managing a child’s asthma as part of daily life | N=15 mothers of children with asthma | A descriptive phenomenological study, using thematically analysed qualitative interviews, followed by focus groups to verify interview findings. |
| Horner, (1998) [27] | USA (rural and urban) | An exploration of how families with school-aged children with asthma assimilate asthma into everyday lives | N=12 families (including n=15 CYP with asthma, aged 6-18 years old)  Nine of the children were described as ‘allergy positive’ pp360 [27] | Qualitative interviews using grounded theory methodology. |
| Stewart *et al,* (2012) [38] | 4 Canadian provinces (mostly urban) | Identify young people’s asthma and allergy support needs, barriers to meeting needs and resources available. Outline preferences for a support intervention | Young people (n=57) responded to questionnaires. Qualitative group interviews were conducted with (n=8) younger adolescents and parents (n=8; 6 mothers, n=2 fathers), meeting eligibility criteria of the scoping review age restrictions. Findings from young adults not meeting the inclusion criteria were not extracted. | Mixed methods, using a non-standardised questionnaire and in-depth group interviews. Thematic content analysis was performed using NVIVO and descriptive statistics were used for quantitative results. However, only the qualitative findings were relevant to the scoping review, therefore quantitative results were not extracted. |
| Van Dellen *et al,* (2008) [33] | Amsterdam, Netherlands | Compare beliefs from families (with a child with asthma) of different ethnicities living in Amsterdam. These included native Dutch, Surinamese, Turkish and Moroccan families | CYP (n=40): Grouped (n=26) were 6-12-year-olds and (n=14) were 13-17-years old), and mothers (n=28) | Focus groups (grouped by ethnicity) were analysed using Kleinman’s theoretical model (1980) [cited by 33]. |
| Parikh *et al,* (2018) [56] | Washington DC, USA | To develop an understanding of the barriers and facilitators for asthma management, from parental perspectives held whilst children are in hospital for asthma care | N=10 parents of (n=12) children, all under the age of 12 years | Qualitative interviews were conducted with parents during their child’s in-patient hospital stay. These were subjected to content analysis and quantitative content analysis (authors [56] cite Krippendorff, 2013). |
| Edgecombe *et al,* (2010) [37] | Southampton, Portsmouth, & Isle of White, UK | To understand young people’s experience of living with difficult, severe asthma, with interest in health care professional interactions and medication adherence | (N=22) young people, aged 11-18-years-old | Qualitative semi-structured interviews were analysed using the thematic approach of continual comparison. |
| Trollvik *et al,* (2011) [55] | Norway (mostly rural-(n=12 of n=15 participants) | Explore children’s experiences of asthma to inform the design of an asthma learning programme | Children (n=15), aged 7-10 years  (All had co-existing allergies) | Semi-structured qualitative interviews combined with a meta-communication technique (children drew pictures to highlight asthma experiences and explained these to the researcher) was used. Phenomenological analysis of data was conducted. |
| Lakhanpaul *et al,* (2017) [46] | London, UK | Explore and compare asthma perceptions and experiences between white British and South Asian families. This was planned to identify barriers to management to inform management and interventions that are culturally suitable | Matched (where possible) white British families (n=14 families) and British South Asian families (n=30 families) | Parents, children, and some extended family-members participated in qualitative semi-structured interviews, analysed with interpretive thematic analysis. |
| Raymond *et al,* (2012) [52] | USA | Create a list of asthma management strategies adopted by parent-caregivers (by topic).  Establish whether themes related to asthma severity, lung function and quality of life | Parents of children with asthma, aged 5-12 years (n=200 caregivers) | Mixed methods: qualitative interviews were analysed using open coding and line by line content analysis. Quality of life measures were used for the quantitative component and as results cannot be used to answer the scoping review questions, these were not extracted. |
| Yonas *et al,* (2017) [49] | Pittsburgh, USA | To define experiences and issues in an economically underserved group with asthma. This was designed to inform a community-based intervention. | Parents (n=14) and CYP (n=7) with asthma, living in the Homewood area (considered socio-economically disadvantaged) | Community-based participatory research, using three concept mapping (focus group style) sessions. |
| Hughes *et al,* (2017) [47] | Ireland | Explore young peoples’ asthma in everyday life and develop a theory explaining how young people resolve concerns | N=51 young people, aged 11-16 years | Grounded theory design. Data from 18 interviews and 5 participant diaries (recorded over 2-week period) and 33 asthma consultations were analysed. |
| Mansour *et al,* (2000) [28] | USA (inner city) | Explore parents’ perspectives on the barriers to asthma care in an inner-city, minority group (all parents described their ethnicity as black) | N=40 parents (of n=47 children, aged 5-12 years), with asthma | Focus groups using open-ended facilitator questions. Statistical software was used to count the frequency of barriers mentioned. |
| Jan *et al,* (2014) [42] | Hualien, Taiwan | Develop an understanding of family self-management experiences for children with moderate-severe asthma | Parents only (n=15) of children aged 8-12 years with asthma | Qualitative in-depth interviews were content analysed. |
| Martin *et al,* (2010) [40] | Chicago, USA | Outline family self-management behaviours and beliefs in Midwest Puerto-Rican families with a CYP with asthma | N=32 participants included  Parents (n=13) and CYP, (n=19) aged 9-18 years old | Participatory design using qualitative interviews and focus groups. Analyses were performed following naturalistic inquiry methods. |
| Biksey *et al,* (2011) [39] | Pennsylvania, USA | Determine the extent of parents’ knowledge about environmental asthma risks in homes, their behaviour, willingness to make behavioural changes, and determine any racial differences | (N=12) Parents only (of 4–8-year-olds with asthma): N=8 white families and (n=4) African American families | Mixed methods pilot study. Qualitative interview transcripts were initially coded into themes and a codebook was developed (authors [39] cite Miller & Crabtree, 1992). Transcripts were coded a second time to explore racial differences. Quantitative methods were less clearly outlined. Participants were asked about whether they knew or did not know environmental asthma risks in their homes, and their related behaviours. |
| Yinusa-Nyahkoon*, et al,* (2010) [36] | Boston (inner city), USA | Outline ecological and social barriers families experience in managing children’s asthma | (N=19) African American parents of 5-12-year-olds with asthma | Semi-structured interviews using a constructivist grounded theory approach to qualitative analysis were used Follow up interviews were conducted 1 year later in n=11 cases). |
| Gibson-Scipio *et al,* (2013) [41] | Urban Detroit, USA | Identification of asthma management goals, beliefs and issues facing carers of African American and mixed-race teenagers with asthma | (N=14) caregivers of 14–18-year-old African American or mixed race CYP with asthma | One Focus group was conducted. Content was iteratively analysed, and descriptive themes were outlined. |
| Gibson-Scipio *et al,* (2015) [45] | Urban Detroit, USA | Exploration of management goals, beliefs, and behaviours of African American teenagers with asthma | (N=13) CYP aged 14–18-years-old | One focus group was conducted. A modified grounded theory approach was used. |
| Soo & Tan (2014) [43] | Singapore | An exploration of carers’ knowledge, understanding and perceptions of asthma and how these influenced self/family asthma management | N=14 carers (of CYP with asthma, aged 4-15 years) in 3 focus groups (included 3 ethnicities and n=13 mothers and n=1 father) | Three focus groups were conducted. Content analysis was undertaken with NVIVO software. |
| Mammen *et al,* (2018) [51] | Upstate New York, USA | Develop an understanding of teenage asthma self-management and teen and carers’ perspectives on the important aspects of self-management | (N=28 participants / 14 family dyads including parents and CYPs). Teens were aged 13-17 years. Most were categorised as having moderate asthma, but some had mild or severe asthma | Qualitative interviews with CYP and parents were conducted, and CYP voice diaries recordings were collected. Data analysis followed the 3-step process outlined by Walker and Avant [cited by 51] for theory synthesis. After initial analysis, priori codes from Mammen and Rhee’s 2012 [cited by 51] study with clinicians and researchers, were used for comparison (transcripts were re-coded with priori concepts from the previous study. Atlas.ti and Xmind were used for analysis. |
| Shegog *et al,* (2012) [59] | Urban, USA | To classify what CYP and parents believed they could attribute successful or failed asthma self-management tasks/outcomes to | (N=65) CYP (9-13 years, 63% male) and their primary carer participated.  (50.7% of CYP had mild asthma, 38.5% moderate and 10.7% severe) | Participants were randomly presented with scenarios (showing both successful and poor asthma self-management) to facilitate structured interviews. Interview findings were coded by causal dimensions (internal/external; stable/unstable; controllable/uncontrollable) and correlations were calculated to explore relationships between child-parent causal dimensions per each self-management ‘domain’ (domains included: medication adherence, symptom monitoring and environmental trigger avoidance) [59, pp.274]. |
| Pradel *et al,* (2001) [29] | USA (North Carolina) | Explore knowledge and beliefs about asthma, child’s autonomy in asthma self-care, medication, and any variation in this by age | (N=32) children aged 7-12 years with moderate-severe asthma | Two interview styles were used to triangulate qualitative findings using content analysis  1. An ‘ethnographic’ interview where children were asked to draw the last time they were unwell and then talk about it in interview.  2. An ‘asthma figurative process interview’ (29; pp.201), during the same visit as interview 1, or in a 2^nd^ visit (30 interviews) was used. The aim was for children to describe the processes involved in an asthma exacerbation. Data were coded and Stata 4 for Windows database was used to count the frequency of answers. |
| Prout *et al,* (1999) [58] | North Midlands, England, UK | Explore the adaption perspective in families with cases of childhood asthma | N=9 families with a child (7-12 years) with doctor diagnosed, moderate asthma | Semi-structured interviews were conducted with parents and separately with children. The study included children’s drawings to facilitate discussion and parents made a timeline of events preceding the child’s last asthma exacerbation. Findings were reported per family rather than collectively or thematically. The methodology and methodological references were not explicitly outlined. The authors noted that the  *“study reported in this paper was originally conceived of as broadly within the adaptation*  *Framework”* [58, pp.142]. |
